# The impact of hypothetical PErsonalised Risk Information on informed choice and intention to undergo Colorectal Cancer screening colonoscopy in Scotland (PERICCS)—a randomised controlled trial

**DOI:** 10.1186/s12916-020-01750-3

**Published:** 2020-10-20

**Authors:** Jayne Digby, Ronan E. O’Carroll, Julie A. Chambers, Robert J. C. Steele

**Affiliations:** 1 Centre for Research into Cancer Prevention and Screening, University of Dundee, Ninewells Hospital and Medical School, Dundee, DD1 9SY Scotland, UK; 2 Division of Psychology, University of Stirling, Stirling, FK9 4LA Scotland, UK

**Keywords:** Informed choice, Personalised risk, Faecal immunochemical test, Colorectal cancer screening

## Abstract

**Background:**

There is currently no existing evidence on the effects of personalised risk information on uptake of colonoscopy following first line screening for colorectal cancer. This study aimed to measure the impact of providing risk information based on faecal haemoglobin concentration to allow a fully informed choice around whether or not to undergo colonoscopy.

**Methods:**

Two thousand seven hundred sixty-seven participants from the Scottish Bowel Screening Programme (SBoSP) database, who had not recently been invited for screening, were randomised to receive one of three types of hypothetical risk information materials: (1) numerical risk information (risk categories of one in 40, one in 1600 and one in 3500), (2) categorical risk information (highest, moderate and lowest risk), or (3) positive screening result letter (control group). The primary outcome was the impact of the risk materials on intention to undergo colonoscopy, to allow comparison with the current colonoscopy uptake of 77% for those with a positive screening result in the SBoSP. Secondary outcomes were knowledge, attitudes and emotional responses to the materials.

**Results:**

Four hundred thirty-four (15.7%) agreed to participate with 100 from the numerical risk group (69.0%), 104 from the categorical risk group (72.2%) and 104 from the control group (71.7%) returning completed materials. Intention to undergo colonoscopy was highest in the highest risk groups for the numerical and categorical study arms (96.8% and 95.3%, respectively), but even in the lowest risk groups was > 50% (58.1% and 60.7%, respectively). Adequate knowledge of colorectal screening and the risks and benefits of colonoscopy was found in ≥ 98% of participants in all three arms. All participants reported that they found the information easy-to-understand. 19.1%, 24.0% and 29.6% of those in the numerical, categorical and control group, respectively, reported that they found the information distressing (*p* > 0.05).

**Conclusions:**

Applying the risk categories to existing SBoSP data shows that if all participants were offered an informed choice to have colonoscopy, over two thirds of participants would intend to have the test. Equating to an increase in the number of screening colonoscopies from approx. 14,000 to 400,000 per annum, this would place an unmanageable demand on colonoscopy services, with a very small proportion of cancers and pre-cancers detected. However, the response to the materials were very positive, suggesting that providing risk information to those in lowest and moderate risk groups along with advice that colonoscopy is not currently recommended may be an option. Future research would be required to examine actual uptake.

**Trial registration:**

Date applied 1 December 2017 ISRCTN number 14254582.

## Background

Following a successful demonstration pilot [1] a quantitative faecal immunochemical test for haemoglobin (FIT) was introduced as the primary test in the Scottish Bowel Screening Programme (SBoSP) in late 2017, replacing the guaiac faecal occult blood test (gFOBT). The advantages of FIT over gFOBT are well accepted, including being more user-friendly, specific for human haemoglobin, and importantly, providing an estimation of faecal haemoglobin concentration (f-Hb) rather than a binary positive/negative result. Since f-Hb is related to severity of disease [2], the numerical FIT result is an important risk factor for colorectal cancer (CRC). Indeed, a recent study in a symptomatic population referred for colonoscopy identified f-Hb as by far the most powerful predictor of all risk factors studied including age, gender, symptoms, family history and lifestyle factors [3]. However, in the SBoSP, FIT is currently used as dichotomous test, with the threshold for a positive result set at f-Hb of ≥ 80 μg Hb/g faeces to mimic the 2% positivity rate observed with the previous gFOBT-based screening algorithm. Although not as strongly predictive as f-Hb, increasing age and male sex have also been documented to show association with CRC prevalence [4]. Therefore, there is potential for f-Hb, along with age, sex and other risk factors to be used to provide a personalised risk of harbouring CRC, and thus empower people to make a truly informed decision about having a colonoscopy, by weighing the risks and disadvantages of the procedure (e.g. bleeding, bowel wall perforation, emotional distress) against their personalised risk of missing a cancer. This is particularly important in CRC screening, as currently about half of all cancers in the screened population in Scotland are diagnosed in the interval between screens, indicating that, at the current threshold, the test is only about 50% sensitive for CRC. Reducing the threshold would result in fewer cancers being missed but would increase the chance of a negative colonoscopy and the impact on the colonoscopy service may be unsustainable.

There is currently no existing evidence on the effects of personalised risk information on uptake of colonoscopy following first line screening for CRC. A Cochrane systematic review examined the effect of personalised risk communication for informed decision-making in uptake of medical screening compared to general information [5]. Overall, providing a numerical risk score or a categorised risk (e.g. ‘low’, ‘medium’, ‘high’) increased informed choice (odds ratio 3.65, 95% CI 2.13 to 6.23, for random effects) and screening uptake (odds ratio 1.15 95% CI 1.02 to 1.29). However, the included studies covered a wide range of screening tests and thus the results were heterogeneous. Of the studies involving CRC, only one [6] used a calculated numerical risk score, leading to greater knowledge but non-significant *lower* intention and uptake; it did not report changes in informed choice. Three studies used a categorised risk score; these did not assess knowledge or informed choice, but indicated a small, significant increase in uptake of screening [7–9]. The authors concluded that the evidence that personalised risk communication increases screening uptake is weak. Further, although some included colonoscopy, all of the reviewed studies involving CRC screening related to first-line screening only. Subsequent to the commencement of our study, results have been published from the CRISP-Q [10] study where patients in GP waiting rooms in Australia were given five different hypothetical average and increased risk presentations and asked to decide for each, based on the risk presented, whether they would choose either no screening, a faecal occult blood test, or a first-line screening colonoscopy. Trends existed for selection of more appropriate screening options both for the risk presentation showing an absolute risk of CRC along with a government recommendation and the one showing an ‘expected frequency tree’. Very recently published results from a Danish RCT found that a web-based decision aid aimed at screening-naïve individuals with lower educational attainment did not affect informed choice or knowledge of CRC screening but there were trends towards improved uptake and positive attitudes towards CRC screening compared with the control group [11].

Currently, participants in the SBoSP are offered colonoscopy when they have f-Hb ≥ 80 μg Hb/g faeces. This threshold for positivity is much higher than that used in other countries. Any measurable f-Hb confers some risk of neoplasia, and sensitivity for cancer detection increases as the f-Hb threshold is lowered [12]. It is likely that those screening participants receiving a letter informing them that they have had a negative screening test result are largely unaware that around half of all CRC in the screened population arise in those with f-Hb below the threshold of 80 μg Hb/g faeces. Thus, we aimed to investigate the impact of providing the screened population with novel, hypothetical personalised risk information designed to enable a completely transparent informed choice as to whether or not to undergo colonoscopy following screening with FIT. In addition to the effect on level of informed choice, we also investigated the potential impact that providing screening participants with such information would have on colonoscopy services as well as emotional responses such as increased anxiety about the screening process.

## Methods

### Setting

The National Health Service (NHS) in Scotland provides comprehensive health care to all permanent residents, funded by general taxation but free at the point of use, based on need. Currently in Scotland, all men and women aged 50–74 years and registered with a NHS general practitioner (GP) practice are invited to participate in bowel screening every 2 years. About 850,000 invitations are sent each year via the Bowel Screening Scotland information technology system (BoSS), which holds a database of all eligible participants. The most recently published Key Performance Indicators report uptake of FIT in the SBoSP at 63.9%, with a positivity rate of 3.1%. Colonoscopy uptake in those with a positive screening test (f-Hb ≥ 80 μg Hb/g faeces) was reported as 77.3%.

### Design

The full study protocol has been published previously [13]. Novel personalised risk information materials (both for risk of having CRC and the risks associated with colonoscopy) were developed for a simple three arm randomised controlled trial (RCT) design. The three study arms were as follows: (1) numerical personalised risk information, (2) categorical risk information and (3) positive screening test result. Arm 3 was effectively a control group reflecting current practice to allow comparison of likely uptake of colonoscopy with the groups receiving personalised risk information.

The materials were co-developed at workshops each attended by 8 to 12 Patient and Public Involvement (PPI) representatives, the core project team and an infographics expert. The representatives were members of the Health and Behaviour PPI group based at The University of Stirling and were selected to be representative of the screening population. Draft materials were presented at the workshop and completed by the PPI representatives before being reviewed in detail from a lay perspective, with PPI feedback also gathered on the perceived burden associated with receiving and completing the materials. These workshops were audio recorded and the materials were revised according to the feedback provided. Workshops continued to be held until agreement was reached on finalised materials.

A Scenario Letter/s Booklet was developed for each study arm, with risk information for those in the numerical and categorical risk groups provided in a visual format (based on infographics), aimed to be understandable across education levels, as guided by recommendations [14, 15]. For study arms 1 and 2, three hypothetical screening result letters were included in the booklet, each giving a different level of risk of having bowel cancer. In the numerical risk group (Additional file 1), the highest risk letter informed the participant that, based on FIT screening result, age and gender, 1 in 40 people like them would have bowel cancer diagnosed in the next 2 years. This ratio was derived from the number of people with a positive screening test result (f-Hb ≥ 80 μg Hb/g faeces) in the SBoSP pilot demonstration of FIT [1] who had CRC diagnosed in the 2 years following their test result (screen-detected CRC plus interval CRC). The level of risk in the moderate risk letter was 1 in 1600, representing the number of people in the FIT pilot with f-Hb 1 to 79 μg Hb/g faeces who were diagnosed with an interval CRC. The lowest risk provided was 1 in 3500, representing the number of people in the FIT pilot with f-Hb reported as 0 μg Hb/g faeces who had an interval CRC. A green ‘donut’ style infographic with a red segment representing the ratio presented was used for each level of risk. The three letters provided to those randomised to the categorical risk arm (Additional file 2) included a ‘traffic light’ style infographic to convey that the participant was either in the lowest risk group (highlighted in green), moderate risk group (amber) or highest risk (red). A short paragraph explaining what was meant by each risk group was provided below the infographic. Those randomised to the control group received a scenario letter which was representative of the current letter sent to SBoSP participants following a positive bowel screening test based on the cut-off f-Hb of ≥ 80 μg Hb/g faeces (Additional file 3). The negative screening result letter was not provided to the control group since the intention was to mimic current practice, which would not involve this group being offered colonoscopy.

In addition to Scenario letters, the pack which the three groups received in the mail also included two information/education booklets about bowel screening and colonoscopy: the current Bowel Screening Test Booklet (Additional file 4; explaining the benefits of participating in screening as well as explaining that not all CRC are detected by screening) and a Colonoscopy Information Booklet (Additional file 5; based on the materials currently provided to those who are offered colonoscopy following a positive screening test, explaining the procedure including preparation and associated risks). A Study Questionnaire (Additional file 6) was also developed at the PPI workshops and sent to all participants.

The primary outcomes were to assess the impact of different presentations of risk information on informed choice and intention to take up an offer of colonoscopy after FIT. The informed choice measure was adapted from Smith et al. [16] who applied a multidimensional model of informed choice (originally developed and validated for antenatal screening for Down’s syndrome [17, 18]) and combined the constructs of (a) knowledge, (b) attitudes and (c) behaviour to assess the extent to which people made an informed choice about participating in screening using gFOBT.

### Measures

Table 1 summarises the various measures to determine level of informed choice, including intention to accept an offer of colonoscopy, decisional conflict, planned behaviour, knowledge (based on the information provided in the Bowel Screening Test booklet and the Colonoscopy Information Booklet) and attitudes towards bowel screening and colonoscopy. A secondary outcome was to assess participants’ responses to receiving personal risk information. In addition to knowledge and attitudes to screening and risk, this also included emotional responses including anxiety and level of distress felt when reading the materials supplied. Details of these measures are also listed in Table 1.

**Table 1:** Summary of study measures

Measure	Booklet	Repeated measure?	Scoring system	Scoring system and interpretation
Intention to undergo colonoscopy	Scenario Booklet	Yes, following each risk scenario.	Single question to measure intention to accept an offer of colonoscopy as a proxy measure for behaviour if receiving that letter.	N/A.
Decisional conflict	Scenario Booklet	Yes, following each risk scenario.	The informed subscore (items 1–3) from the Decisional Conflict Scale [19] was used to assess the extent to which participants felt informed about their decisions. These items are: “I know which options are available to me”, “I know the benefits of each option” and “I know the risks and side effects of each option”, scored on a 7-point scale from “strongly agree” to “strongly disagree”.	In accordance with scoring system supplied with the tool: responses to each of the three items scored on a scale from 0 to 6 and total score calculated by multiplying the average of the three scores by 16.6 to give a score between 0 and 100. A lower score indicates a more informed choice. Scores lower than 25 are associated with following through with decisions whereas scores greater than 37.5 are associated with decision delay or feeling unsure about implementation of the decision [19].
Planned behaviour	Scenario Booklet	Yes, following each risk scenario.	Two additional 7-point Likert scale responses (from “strongly agree” to “strongly disagree”) to two statements: “If I received information that (1) my risk of bowel cancer was 1 in 40, (2) my risk of bowel cancer was high or (3) the result of the test I provided showed that further investigation is required, then I would intend to have a colonoscopy” and “If I was told that (1) I had a 1 in 40 chance of having bowel cancer, (2) I was in the group at highest risk of bowel cancer or (3) my screening test was abnormal meaning that there was a risk I had bowel cancer, then I would definitely choose to have a colonoscopy”.	Scores ranged from 1 to 7 for each statement, with a higher score representing greater agreeability with the statement.
Knowledge	Questionnaire Booklet	No	Eight questions (four concept, four numerical); 1 point per correct response with the exception the numerical question relating to the number of people surviving bowel cancer if detected early, worth 2 points.	Maximum knowledge score of 9. As with Smith et al. [16] a pass mark of 50% or greater (≥ 5) was used to determine whether or not knowledge was adequate.
Attitudes	Questionnaire Booklet	No	Nine-item scale scored on a 5-point Likert scale from “strongly agree” to “strongly disagree”. Items included discomfort and embarrassment during colonoscopy, side-effects of bowel preparation and beliefs around reducing the risk of dying from bowel cancer.	The range for the total score was 9 to 45. A higher score reflected a more positive attitude towards having a colonoscopy. In keeping with Smith et al. [16], the median value of the sample was used to classify participants’ attitudes towards colonoscopy as being either positive or negative.
Emotional responses	Questionnaire Booklet	No	The questionnaire measured anxiety using a previously-validated six-item version of the State Trait Anxiety Inventory (STAI) [20]. An example item is the statement “I am worried”, with a choice of four responses on a scale from “not at all” to “very much”.	The scoring system was developed to allow comparison with the normative values given for the original 40-item scale [21]. Total possible scores ranged from 20 to 80 with a higher score indicating a higher level of anxiety.
Ease-of-understanding and acceptability	Questionnaire Booklet	No	Two Likert-type questions: “I found the information presented easy to understand” and “I found the information presented distressing” scored on a 7-point scale from “strongly agree” to “strongly disagree”.	Scores ranged from 1 to 7 for each statement, with a higher score representing greater agreeability with the statement.
Previous colonoscopy	Questionnaire Booklet	No	Single question for participants if they had previously had a colonoscopy; Yes, No, or Unsure.	N/A.
Additional thoughts on materials	Questionnaire Booklet	No	Open-ended question encouraging additional free text comments.	Thematic qualitative analysis.

### Pretesting of materials

One hundred forty-four adults registered on the SBoSP database (age range 50 to 74 years), regardless of whether or not they had previously participated in screening, were selected for invitation for pilot testing prior to the materials being refined and finalised for main recruitment. All age quintiles, Scottish Index of Multiple Deprivation (SIMD) categories and both genders were represented in the sample with the target recruitment weighted towards those groups who may be less likely to participate, based on response levels obtained from our large-scale questionnaire study on bowel screening [22], with the aim of achieving a balanced sample of participants. Invitations were not sent to those who had recently participated in screening or were shortly to be invited for screening (within 6 months of either event) to limit any confusion between the study materials and an actual screening result. Invitation letters were sent on behalf of the Principal Investigator, Director of the SBoSP, to ask if they would like to participate in a survey to assess their response to an offer of colonoscopy in relation to an estimated personalised risk of having cancer. A full patient information sheet and a response form were included along with a pre-paid envelope for return. In line with published criteria for increasing recruitment [23], invitation letters were personally addressed, included stamps rather than franked return envelopes and used coloured ink. The response form also included an option to consent to taking part in a short follow-up interview by telephone. On receipt of the returned completed response form, participants were randomised to receive the study materials from one of the three study arms. Blinded randomisation with minimisation on variables related to risk (i.e. age, SIMD, gender) was carried out via MINIM software by an individual independent to the process of running the study. Data on screening history (i.e. previous participation/failure to participate/been offered a colonoscopy) was also collected at the time of sampling, as these strongly predict screening uptake [24]. Return of the completed questionnaire was considered as an implied consent. Of the 144 participants invited for pilot testing, 39 replied with 21 agreeing to take part; 15 completed and returned the study materials. Fourteen out of the 15 participants recorded an adequate knowledge score indicating that the information materials were effective in conveying the relevant facts relating to bowel screening and colonoscopy. The response to the materials was very positive with all participants agreeing that the materials were easy-to-understand. The results of the pilot testing did not indicate a need for any modifications to the methodology for the main recruitment phase of the RCT.

### Main recruitment

Following the pilot testing and finalisation of study materials, a total of 2767 participants were invited to participate in the main study between February and July 2019, following the same process as described for the pilot testing. The required sample size was 300 (100 in each group), calculated to have 83.7% power of detecting a 1 point increase in knowledge (intervention versus control), and a 2-point difference in attitudes (based on existing study means/SDs [16, 17]), using a one-way analysis of variance (ANOVA).

### Procedures

Knowledge, attitudes and intention to take up colonoscopy were compared between the study arms to assess whether providing personalised information on CRC risk led to informed choice. Participants were deemed to be fully informed if they demonstrated adequate knowledge *and* attitudes which match their intention, i.e. a positive attitude to colonoscopy along with the intention to undergo colonoscopy, or a negative attitude to colonoscopy along with the intention not to undergo colonoscopy. A partially informed choice was one where either the participant had adequate knowledge but their attitudes did not match their intention or inadequate knowledge but attitudes matched intention. An uninformed choice was when the participant neither had adequate knowledge or attitudes matching their intention. Between-group differences in outcomes on the scales relating to knowledge, attitudes and behaviour were analysed as continuous variables via ANOVA or Student’s *t* test. The number of participants reporting an intention to accept the offer of colonoscopy from each scenario was used to estimate total uptake, and hence the potential impact on colonoscopy services for numerical versus categorical presentation of personalised risk information, in comparison to the current positive/negative cut-off.

Thirty participants were sampled for semi-structured telephone interview, 10 per arm, from those consenting to be telephoned. All who answered the telephone call agreed to participate and those who could not be reached were replaced in the sample by another consenting participant. Verbal consent was confirmed at the start of the telephone interview, including consent for audio-recordings. The telephone interviews included open-ended questions relating to emotional responses to the materials and understanding of what was meant by the risk categories, as well as asking how participants perceived the risk of undergoing a colonoscopy versus that of having CRC in each of the given scenarios. The recruitment of *n* = 30 to the qualitative interviews was based on the number required for saturation of themes in previous research by the authors; this was also achieved for this sample. Each interview was analysed on a line-by-line basis for key themes/patterns and thematic qualitative analysis was used to compare responses between those who intended and did not intend to take up the offer of colonoscopy. Differences with respect to age, sex, SIMD, and previous CRC screening participation and/or previous colonoscopy were also explored.

## Results

Two thousand seven hundred sixty-seven people were invited to participate in the main study phase, with 434 (15.7%) people indicting they would be willing to participate. Of the 434 people agreeing to participate, 145, 144 and 145 were randomised into the numerical risk arm, categorical risk arm and the positive result letter arm, respectively. Three hundred nine (71.0%) participants completed and returned the materials, 100 from the numerical risk group (69.0%), 104 from the categorical risk group (72.2%) and 104 from the control group (71.7%). Of those who completed and returned the study materials, 91.0%, 93.3% and 90.4% in the respective study arms had previously participated in the SBoSP. Demographic details of those invited and responding to the study invite are shown in Table 2.

**Table 2:** Demographic details of study population

	Number invited (%)	Accepted invite (%)	Returned materials (%)	Numerical risk study arm (%)	Categorical risk study arm (%)	Positive result letter arm (%)
**Gender**						
**Male**	1391 (50.3)	224 (51.6)	154 (50.0)	50 (49.5)	50 (48.1)	54 (52.4)
**Female**	1376 (49.7)	210 (48.4)	154 (50.0)	51 (50.5)	54 (51.9)	49 (47.6)
**Age (years)**						
**50–54**	354 (24.8)	88 (20.3)	57 (18.5)	16 (15.8)	20 (19.2)	21 (20.4)
**55–59**	332 (20.8)	84 (19.4)	52 (16.9)	19 (18.8)	17 (16.3)	16 (15.5)
**60–64**	576 (19.3)	105 (24.2)	76 (24.7)	26 (20.8)	25 (24.0)	25 (24.3)
**65–69**	460 (16.6)	73 (16.8)	59 (19.2)	21 (20.8)	18 (17.3)	20 (19.4)
**70–74**	511 (18.5)	84 (19.4)	64 (20.8)	19 (18.8)	24 (23.1)	21 (20.4)
SIMD* quintile						
**1 (most deprived)**	779 (28.2)	80 (18.4)	51 (16.6)	16 (15.8)	16 (15.4)	19 (18.4)
**2**	623 (22.5)	85 (19.6)	59 (19.2)	20 (19.8)	21 (20.2)	18 (17.5)
**3**	514 (18.6)	83 (19.1)	55 (17.9)	20 (19.8)	17 (16.3)	18 (17.5)
**4**	439 (15.9)	95 (21.9)	72 (23.4)	21 (20.8)	26 (25.0)	25 (24.3)
**5 (least deprived)**	412 (14.9)	91 (21.0)	71 (23.1)	24 (23.8)	24 (23.1)	23 (22.3)

*Scottish Index of Multiple Deprivation

Participants’ responses to whether or not they would choose to have a colonoscopy in each of the risk scenarios are shown in Table 3. In the two study arms receiving risk information, the proportion of participants who said they would intend to undergo colonoscopy decreased as risk level was lowered. This fall in intention was statistically significant in the numerical risk study arm between both of the reductions in risk (1 in 40 cf. 1 in 1600: *p* = 0.0002 and 1 in 1600 cf. 1 in 3500 *p* = 0.003) and in the categorical risk study arm between the moderate and lowest risk scenarios (*p* < 0.0001). Although the number of participants in the categorical study arm who said they would undergo colonoscopy if they were told they were in the moderate risk group was 10% greater than in the equivalent group in the numerical arm (88.8% vs. 78.7%), this difference was not statistically significant (*p* = 0.067). Mean informed subscore of the Decisional Conflict Scale and numbers of participants with scores associated with implementing decisions (scores < 25) for each study arm are also shown in Table 3. No significant differences in mean informed subscore were observed between the study arms.

**Table 3:** Comparison of intention to undergo colonoscopy between risk categories and study arms

	Numerical risk arm	Categorical risk arm	Positive result letter arm	***p*** value
**1 in 40/highest risk/positive result letter**				
Yes	90 (96.8%)	87 (95.3%)	81 (93.1%)	0.493
No	1 (1.1%)	0 (0.0%)	1 (1.1%)	
Unsure	2 (2.2%)	6 (6.5%)	5 (5.7%)	
**1 in 1600/moderate risk**				
Yes	74 (78.7%)	79 (88.8%)	–	0.067
No	5 (5.3%)	1 (1.1%)	–	
Unsure	15 (16.0%)	9 (10.1%)	–	
**1 in 3500/lowest risk**				
Yes	50 (58.1%)	54 (60.7%)	–	0.734
No	12 (14.0%)	15 (16.9%)	–	
Unsure	24 (27.9%)	20 (22.5%)	–	
**Decisional Conflict Scale informed subscale score**				
**1 in 40/highest risk/positive result letter**				
0	56 (60.2%)	66 (71.0%)	60 (68.2%)	
1–25	34 (36.6%)	26 (28.0)	25 (28.4%)	
> 25	3 (3.2%)	1 (1.1%)	3 (3.4%)	
Mean score (95% CI)	6.4 (4.0–8.9)	4.3 (2.8–5.8)	5.7 (3.5–7.8)	0.353
**1 in 1600/moderate risk**				
0	52 (56.5%)	61 (65.6%)	–	
1–25	36 (39.1%)	30 (32.3%)	–	
> 25	4 (4.3%)	2 (2.2%)	–	
Mean score (95% CI)	7.2 (4.9–9.6)	6.4 (3.8–9.0)	–	0.624
**1 in 3500/lowest risk**				
0	50 (55.6%)	54 (58.7%)	–	
1–25	35 (38.9%)	34 (37.0%)	–	
> 25	5 (5.6%)	4 (4.3%)	–	
Mean score (95% CI)	7.8 (5.4–10.1)	8.4 (5.3–11.6)	–	0.732

For the two additional 7-point Likert scale statements regarding intention to undergo colonoscopy (e.g. “If I received information that my risk of bowel cancer was 1 in 40 then I would intend to have a colonoscopy” and “If I was told that I had a 1 in 40 chance of having bowel cancer then I would definitely choose to have a colonoscopy”), all three groups showed a high level of agreement with the statements for the highest risk scenarios (all mean scores ≥ 6.5 out of 7) and even in the lowest risk groups in the numerical and categorical arms, participants still responded positively. Mean scores in each study arm for these items are shown in Table 4.

**Table 4:** Mean scores for additional statements regarding intention to undergo colonoscopy (maximum = 7)

	Numerical risk arm (mean score out of 7, (95% CI))	Categorical risk arm (mean score out of 7, (95% CI))	Positive result letter arm (mean score out of 7, (95% CI))	***p*** value
**“If I received information that my risk of bowel cancer was 1 in** ***x*** **then I would intend to have a colonoscopy”**				
1 in 40/highest risk/positive result letter	6.4 (6.1–6.6)	6.7 (6.5–6.8)	6.5 (6.3–6.7)	0.085
1 in 1600/moderate risk	5.4 (5.1–5.8)	6.4 (6.2–6.6)	–	< 0.0001
1 in 3500/lowest risk	5.0 (4.7–5.4)	5.3 (4.9–5.6)	–	0.3484
**“If I was told that I had a 1 in** ***x*** **chance of having bowel cancer, I would definitely intend to have a colonoscopy”**				
1 in 40/highest risk/positive result letter	6.5 (6.3–6.7)	6.7 (6.6–6.9)	6.7 (6.6–6.9)	0.124
1 in 1600/moderate risk	5.6 (5.2–5.9)	6.4 (6.2–6.6)	–	< 0.0001
1 in 3500/lowest risk	5.0 (4.7–5.4)	5.1 (4.7–5.6)	–	0.6568

Scoring system: “strongly disagree” = 1, “disagree” = 2, “slightly disagree” = 3, “neither agree or disagree” = 4, “slightly agree” = 5, “agree” = 6, “strongly agree” = 7

Mean knowledge and attitude scores for each study arm are shown in Table 5. All participants who completed the knowledge questions (97, 102 and 104 in the numerical risk, categorical risk and positive result letter arms, respectively) recorded a score of ≥ 5, indicating adequate knowledge, with the exception of one in the categorical risk group and two in the positive result letter group. No significant differences in total knowledge or attitude scores were observed between the three study arms (*p* > 0.05). This would be expected as all groups received the same information materials, with only presentation of risk differing. Table 6 shows the number of participants in each study arm who correctly answered each item in the knowledge section of the questionnaire.

**Table 5:** Knowledge and attitude scores and intention to undergo colonoscopy in each study arm

	Numerical risk arm	Categorical risk arm	Positive result letter arm
**Knowledge score (mean)**			
Concept, max. score 4 (95% CI)	3.7 (3.6–3.8)	3.7 (3.5–3.8)	3.6 (3.4–3.7)
Numerical, max. score 5 (95% CI)	4.7 (4.6–4.8)	4.6 (4.5–4.8)	4.5 (4.4–4.7)
Total, max. score 9 (95% CI)	8.4 (8.2–8.6)	8.3 (8.1–8.5)	8.1 (7.9–8.4)
No. with adequate knowledge (score ≥ 5)	96/97 (99.0%)	100/102 (98.0%)	102/104 (98.1%)
**Attitude**			
Mean score (95% CI)	38.3 (37.3–39.4)	38.8 (37.9–39.7)	38.7 (37.6–39.8)
No. with positive attitude towards colonoscopy (score ≥ 39)	48/93 (51.6%)	59/97 (60.8%)	63/101 (61.4%)

**Table 6:** Proportion of participants correctly answering each knowledge question in each study arm

	Numerical risk arm	Categorical risk arm	Positive result letter arm	***p*** value
**Q1:** Can people without symptoms have bowel cancer? (Y/N)	94/97 (96.9%)	100/102 (98.0%)	103/104 (99.0%)	0.556
**Q2:** How many people out of 10 survive bowel cancer if treated early? (4/6/9)	88/97 (90.7%)	92/102 (90.2%)	94/104 (90.4%)	0.992
**Q3:** Do you think that all cancers bleed, meaning that the bowel cancer screening test will find every cancer? (Y/N)	93/97 (95.9%)	101/102 (99.0%)	101/104 (97.1%)	0.378
**Q4:** The bowel preparations required before a colonoscopy will mean you experience urgency to go to a bathroom and you will need to remain at home (Y/N)	87/97 (89.7%)	91/102 (89.2%)	90/104 (86.5%)	0.750
**Q5:** How long does a colonoscopy take? (5–10 min/20–45 min/1–2 h)	90/97 (92.8%)	93/102 (91.2%)	95/104 (91.3%)	0.903
**Q6:** How many people who have a colonoscopy will experience bowel perforation (tear of the lining of the bowel)? (1 in 2000/1 in 500/1 in 200)	95/97 (97.9%)	97/102 (95.1%)	89/104 (85.6%)	0.002
**Q7:** Bleeding occurs with approximately one in every 100–200 polyp removals (true/false)	92/97 (94.8%)	93/102 (91.2%)	93/104 (89.4%)	0.365
**Q8:** The sedation used during colonoscopy occasionally cause problems with breathing, heart rate and blood pressure (true/false)	89/97 (91.8%)	89/102 (87.3%)	86/104 (82.7%)	0.159

For the highest risk groups only (risk of 1 in 40 in the numerical group, highest risk in the categorical group and the positive result letter group), Table 7 shows the number of participants in each study arm who were deemed to have made a fully informed, partially informed or uninformed choice about whether or not to have a colonoscopy. One person in the numerical study arm and one person in the positive result letter study arm made a fully informed choice not to have a colonoscopy, while the remainder of those who were classed as fully informed said they would take up the offer of colonoscopy. No significant difference was found when comparing the level of informed choice between the three study arms (*p* > 0.05).

**Table 7:** Comparison of level of informed choice between study arms

	Numerical risk arm (1 in 40)	Categorical risk arm(highest risk)	Positive result letter arm
Fully informed choice*	46/85 (56.5%)	54/81 (65.4%)	51/80 (63.8%)
Partly informed choice**	38/85 (44.7%)	28/81 (34.6%)	28/80 (35.0%)
Completely uninformed choice***	0/85 (0.0%)	0/81 (0.0%)	1/80 (1.3%)

*Adequate knowledge + positive attitude + decision to undergo colonoscopy OR adequate knowledge + negative attitude + decision not to undergo colonoscopy

**Adequate knowledge + negative attitude + decision to undergo colonoscopy OR inadequate knowledge + positive attitude + decision to undergo colonoscopy

***Inadequate knowledge + negative attitude + decision to undergo colonoscopy OR *** inadequate knowledge + positive attitude + decision not to undergo colonoscopy

The mean anxiety score for those in the positive result letter arm (36.8, 95% CI 33.9 to 39.8) was statistically significantly higher than for those in the numerical study arm (32.2, 95% CI 29.8 to 34.6, *p* = 0.02) and those in the categorical study arm (33.8, 95% CI 30.1 to 35.4, *p* = 0.04).

The proportion of subjects who reported that they strongly agreed with the statement that the information was easy to understand was 61.2%, 72.5% and 66.3% in numerical risk group, the categorical risk group and positive result letter group, respectively. The differences between the study arms were not statistically significant (*p* > 0.05). No participants across any of the study arms showed any disagreement with this statement. 19.1% of those in the numerical risk group, 24.0% of those in the categorical risk group and 29.6% of those in the positive result letter group agreed to some extent that they found the information presented distressing (*p* > 0.05), although less than 5% in each arm strongly agreed.

One hundred twenty-four (40.3%) of all participants reported that they had previously had a colonoscopy, with 43 (43.0%) in the numerical risk study arm, 41 (39.4%) in the categorical risk arm and 40 (38.5%) in the positive result letter arm. Logistic regression analysis showed that previous experience of colonoscopy did not appear to have an impact on intention in the highest and moderate risk groups for the two risk information groups or for the positive result letter study arm (*p* > 0.05). In the lowest risk group in the categorical risk study arm, however, people were more likely to report that they would accept the offer of a colonoscopy if they had previously had the test (odds ratio [OR] 3.13, 95% CI 1.20 to 4.02). Overall, previous colonoscopy experience was associated with a positive score for attitude towards bowel screening and colonoscopy (OR 1.81, 95% CI 1.11 to 2.95), but not when assessed for the individual study arms (*p* > 0.05). Previous experience of colonoscopy did not show association with STAI anxiety scores. Even when classifying participants according to a STAI score cut-off of > 40 (considered to indicate clinically significant symptoms of anxiety [25]), the ORs to indicate a relationship with previous colonoscopy experience were not statistically significant for any of the three study arms, nor overall (*p* > 0.05).

Thirty participants took part in telephone interviews, with 10 from each of the three study arms. All participants across all groups said that they found the scenario letters easy-to-understand acceptable and would not put them off from future participation in the screening programme. In general, participants had a good understanding of how increasing age and the amount of blood found in the screening test sample act as risk factors for CRC, although many were not clear on male sex as a risk factor. Participants in the numerical and categorical risk information groups were asked about their understanding of the different risk groups they were presented with, their reasons for deciding whether or not they would accept the offer of colonoscopy and how they felt the risks of the procedure compared to the benefits. Of those who completed a telephone interview, four participants in the numerical risk information group and six participants in the categorical risk information group had indicated on their returned materials that they would accept the offer of colonoscopy in all three risk scenarios. Explanations included “Although the medium and low scenarios had a much reduced chance of developing bowel cancer, if I was offered a colonoscopy I would accept, as I wouldn’t think it was being offered unless there was justifiable reason for doing the procedure” (numerical risk group) and “For peace of mind, even although you were low risk you would say ‘let’s know for certain’” and “I feel that if there was even the slightest risk of developing bowel cancer, the discomfort of a colonoscopy is a small price to pay for early detection” (categorical risk group). Two participants in the numerical risk group reported that they would like to be told either way whether or not they should have a colonoscopy, with one saying “I would like to know ‘yes’ you have bowel cancer or ‘no’ you do not have bowel cancer. I feel that telling me I have a 1 in 400 or 1 in 4000 chance of getting bowel cancer is as much use to me as being told my chances of dying as a result of a car crash or being hit by a bus; just something else to worry about!”. Conversely, two participants in the categorical risk group reported that they would like to be provided with a numerical level of risk, saying “I feel if percentages of risk and recovery were given, I could have made a more informed decision” and “I would have appreciated more hard facts at this stage, actual percentages of those in each of the risk groups subsequently found to have bowel cancer”. The majority of participants across all three study arms expressed that they felt that the risks associated with colonoscopy were outweighed by the benefits of having a colonoscopy. No differences in themes identified in the qualitative data were found between different age quintiles, sexes or SIMD quintiles.

The questionnaire also allowed participants to record their views on the information presented using free text. In addition to the participants who completed telephone interviews, a further 172 patients (59, 64 and 49 in the numerical risk arm, categorical risk arm and positive result letter arm, respectively) took this opportunity. Twenty-eight (47.5%), 18 (28.1%) and 30 (61.2%) participants, respectively in each study arm, made comments giving positive feedback regarding the ease of understanding and acceptability of the materials provided. Participants in the risk information arms also spoke positively about making an informed choice. One participant in the numerical risk arm said “Though I would be concerned about any future risk of cancer, I prefer an honest approach to the situation”*.* Similarly, those in the categorical risk arm wrote comments such as “Gave me a clear understanding i.e. risk. Would prefer to know as much as possible of my situation and risk level”. However, one participant in the categorical risk arm said, regarding a previous screening experience, “I didn’t receive a booklet regarding risks - glad I didn’t, I now feel more anxious and concerned if I need a third colonoscopy! Sometimes ignorance is bliss!” and another in the positive result letter arm said “Some people will think it’s embarrassing or worry too much given too many details”. A few participants commented on the use of colours in the infographics, with one participant saying it was helpful (categorical risk group) while one in each of the risk information groups said the use of red was *“frightening”*. Some participants in the categorical risk arm gave feedback that they were not clear on the levels of risk provided to them, for example “I think I understand what is meant by ‘lowest risk’ and ‘highest risk’, but ‘moderate risk’ is more difficult. Can you provide more information about this category? I think I would err on the side of caution and have a colonoscopy - if only to reduce ‘moderate risk’ to ‘lowest risk’” and “The letters would suggest everyone, regardless of risk will be offered a colonoscopy. However, if there is a “not currently at risk” group then this should be incorporated”. Three participants also commented that their responses to the materials may have been different if they received them in real life, rather than hypothetically.

## Discussion

When given the choice whether or not to have a colonoscopy, the majority of participants reported that they would take up the offer, regardless of the level of risk that they are provided with. Responses to the open-ended questions in the questionnaire and during telephone interviews demonstrated a theme that many people consider the risk of CRC as being more serious than the risks of colonoscopy, even when the latter is statistically more likely. Existing data on FIT in the SBoSP indicates that 2.5%, 38.8% and 58.7% of people would be in the highest, moderate and lowest risk groups, respectively. Combining these proportions with the findings of this study suggests that if all bowel screening participants were offered an informed choice to have colonoscopy, over two thirds of participants would opt to have the test. This would represent an increase in the current number of screening colonoscopies performed in Scotland from approx. 14,000 per annum to approx. 400,000 per annum. Clearly, an additional demand of this magnitude on colonoscopy services would not be manageable, with only a very small proportion of cancers and pre-cancers detected.

There are currently no accepted measures of informed choice in CRC screening. Our study has shown that bowel screening participants generally respond very positively to receiving personalised risk information using infographics. An encouraging finding was that providing more information does not appear to make people more anxious, with mean anxiety scores lower in those in the groups receiving risk information compared with the control arm. The proportion of participants who reported decisional conflict was very low in both groups who received risk information, for all three levels of risk. However, responses to the intention question used as a proxy for behaviour showed a greater level of uncertainty was apparent with the lowest risk groups (1 in 3500 or lowest risk); over a fifth of participants reported that they were unsure whether or not they would decide to have a colonoscopy. The finding that those at lowest risk were not adequately reassured that colonoscopy was not required indicates that providing participants with a fully informed choice in this setting may be inappropriate if it is not accompanied by some form of advice. Similarly, the qualitative results revealed that there was some uncertainty around the meaning of the categorical risk groups, with ‘moderate risk’ in particular perhaps lacking clarity. This feedback also suggests that providing risk information alongside more explicit advice on whether or not colonoscopy is required is likely to be a more pertinent approach. This may also have the desirable effect of increasing colonoscopy uptake in those at highest risk above its current rate of 77%. The majority of participants in the two risk information groups were classified as being fully informed, although this was similarly true of the positive result letter study arm; being representative of current practice, this is reassuring.

A major strength of the study is that development of the study materials was achieved using iterative feedback provided by a PPI group who were selected to be representative of the population eligible for bowel screening in Scotland. They unanimously agreed on the layout, content and readability of the final materials giving the study team confidence that the materials would provide an informed choice about whether or not to undergo a colonoscopy. Indeed, the vast majority of participants in all study arms and across all risk categories recorded scores on the informed subscore of the Decisional Conflict Scale which are associated with implementing decisions. In addition, final recruitment of participants was positive, with targets of 100 in each study arm being met. A further strength is that study invitations were weighted towards those in demographic groups previously shown to be least likely to participate. Although participation was still lowest in those in the most deprived quintiles, this would have been more pronounced without the selective invitation process. Comparable scores were recorded across the three study arms for knowledge of CRC screening and colonoscopy. This would be expected since all three received the same information materials, but it is reassuring that a similar, high level of understanding was demonstrated by the three groups.

The main limitation of the study is we cannot account for the likely gap between intention and behaviour. Indeed, free text comments in the study questionnaire included indications that the thought process when deciding whether or not to undergo colonoscopy may be different in a “real life” scenario. To fully assess the impact of offering a more informed choice on uptake of screening colonoscopy would require incorporation of a full scale RCT into the screening programme invitation process. However, the results of this study suggest that even with adjustment for the intention-behaviour gap, a huge increase in demand on the colonoscopy resource would still occur.

Since the study invitation was separate to the actual screening invitation, with the cohort including people who had not previously taken part in screening, the results are applicable to the overall population eligible for screening. However, a further potential limitation of the study is participant bias, in that those who accepted the study invitation and then completed and returned the materials are those who are more likely to be more health conscious and participate in screening. However, in actual practice, the offer of informed choice following FIT would only be made to those who had completed the test, so we believe this would be an acceptable, if not desirable, bias. The finding that 40% of participants had previously had a colonoscopy may also represent participation bias. It is likely that a reasonable proportion of the screening population as a whole, given the age range, will have had previous experience of colonoscopy but we do not have data available for comparison to measure the significance of this as a study limitation.

Numerical risks were calculated according to three subgroups of f-Hb only. In reality, the level of risk given would be further refined according to age and gender. The use of only three numerical risk levels (1 in 40, 1 in 1600 and 1 in 3500) allowed intra-participant comparison between all responses to the three scenarios in this study arm. For the same reason, all participants in the numerical and categorical risk arms received all three levels of risk within their scenario booklet (1 in 40, 1 in 1600 and 1 in 3500 or highest, moderate and lowest risk, respectively). However, this opportunity to consider each risk scenario by contrasting with the other two may have had an effect on their reported intention. Therefore, intention may differ if providing personalised risk in reality, where participants only receive one level of risk.

It should also be noted that was only possible to present risk scenarios using existing SBoSP data from the numbers of screen-detected and interval cancers. Since the screening programme aims to detect both CRC and its pre-cursors, the calculated risk scenarios would be an underestimate of the risk of all screen-relevant lesions.

Some participants reported that they would like to consult a medical professional about their decision whether or not to accept the invitation for a colonoscopy. Indeed, a new BMJ guideline for CRC screening [26] is the first to avoid a blanket recommendation for CRC screening in those above a certain age and instead suggests a personalised and risk-based approach should be made between the individual and the clinician. The guideline recommends that using a shared decision tool such as the QCancer® calculator (qcancer.org/15yr/colorectal/) which incorporates age, sex, ethnicity, BMI, smoking status and family history of gastrointestinal cancer to estimate a personal CRC risk over the next 15 years.

Another theme identified in the qualitative analysis was that people thought that colonoscopy would not be offered to them unless it was required. The trust that the UK population holds in the NHS may be a factor contributing to the majority of people reporting that they would intend to have a colonoscopy if it was offered, regardless of risk. If the same protocol was followed in other countries, different results may occur with different healthcare systems to that of the UK. Furthermore, it is important to note that the f-Hb threshold of 80 μg Hb/g faeces used for positivity in the SBoSP is much higher than that used in other countries. Therefore, if a similar approach to ours was adopted in other countries, the moderate risk scenario would cover a much narrower f-Hb range than that of 1 to 79 μg Hb/g faeces in this study. Given our results, it is possible that the blanket offer of colonoscopy to all screening participants may contradict the key principles of screening laid out in the seminal work by Wilson and Jungner [27] and the UK National Screening Committee [28]. These principles outline that in screening programmes the costs and risks (physical and psychological) should be balanced against the benefits and that a suitable cut-off should be defined and agreed. Alternative approaches to improve transparency of information offered to screening participants could also be explored, such as providing those with a negative result with their numerical f-Hb, so that they themselves are aware of the proximity of their screening result to the threshold. This idea is supported by the findings of a recent study from Italy demonstrating that those with two consecutive negative screening test results, but a *cumulative* f-Hb above the cut-off for positivity had a significantly increased risk of a future diagnosis of advanced neoplasia [29]. An indication of the individual’s proximity to the positivity threshold could be provided alongside strategies applying more intelligent use of FIT, such as varying the length of the screening interval according to f-Hb, with those closest to the threshold offered the opportunity to be screened more regularly than those with very low or undetectable f-Hb.

## Conclusions

With a growing interest in informed choice based on levels of risk in screening programmes, our finding that offering fully informed choice may not be feasible when colonoscopy capacity is limited is applicable to CRC screening programmes worldwide. However, the response to the materials was very positive, suggesting that offering risk information to those in the lowest and moderate risk groups along with advice that it is not recommended that they undergo colonoscopy at this time may be an option. A further full-scale RCT would be required to examine the actual uptake of colonoscopy in reality.

## Supplementary information

**Additional file 1.** Scenario letters booklet for numerical risk study arm; shows three imaginary bowel screening result letters with a numerical risk of having colorectal cancer, along with some short questions to complete.

**Additional file 2.** Scenario letters booklet for categorical risk study arm; shows three imaginary bowel screening result letters with a categorical risk of having colorectal cancer, along with some short questions to complete.

**Additional file 3.** Scenario letters booklet for control group (positive result letter) study arm; shows the current letter sent to Scottish Bowel Screening participants following a positive screening result.

**Additional file 4.** Bowel Screening Test Booklet; the current leaflet provided to Scottish Bowel Screening Participants at the time of screening invitation to explain the benefits of participating in screening.

**Additional file 5.** Colonoscopy Information Booklet; contains information to explain to participants what would happen before and during a colonoscopy and the risks and complications associated with the test.

**Additional file 6.** Questionnaire Booklet; contains some further questions on participants’ thoughts on having a colonoscopy and on the information provided to them.

**Additional file 7.** Internal reliability of study measures; contains the results of statistical tests to measure the internal reliability of several of the study measures.

## Data Availability

The datasets used and/or analysed during the current study are available from the corresponding author on reasonable request.

## Abbreviations

ANOVA: Analysis of variance
CI: Confidence interval
CRC: Colorectal cancer
f-Hb: Faecal haemoglobin concentration
FIT: Faecal immunochemical test for haemoglobin
gFOBT: Guaiac faecal occult blood test
OR: Odds ratio
PPI: Patient and Public Involvement
RCT: Randomised controlled trial
SBoSP: Scottish Bowel Screening Programme
SIMD: Scottish Index of Multiple Deprivation
