# Supplementary material for: The impact of hypothetical PErsonalised Risk Information on informed choice and intention to undergo Colorectal Cancer screening colonoscopy in Scotland (PERICCS)—a randomised controlled trial
Source: BMC Med. 2020 Oct 20;18:285. doi: 10.1186/s12916-020-01750-3 (PMC7574531; doi:10.1186/s12916-020-01750-3)
Supplement: Supplementary file 1 — Additional file 1. Scenario letters booklet for numerical risk study arm; shows three imaginary bowel screening result letters with a numerical risk of having colorectal cancer, along with some short questions to complete. [file 12916_2020_1750_MOESM1_ESM.pdf]

STUDY ID: _ _ _ _

Scenario Letters

Impact of risk information in the Scottish Bowel Screening Programme

- In this booklet you will find three different hypothetical bowel cancer screening result letters.
- Each describes a different level of risk of having bowel cancer following completion of a bowel cancer screening test kit.
- Please answer the questions which follow each letter, then complete the separate Questionnaire Booklet.

University of Dundee

NHS SCOTLAND

UNIVERSITY of STIRLING

PRIVATE & CONFIDENTIAL

Scottish Bowel Screening Centre
Kings Cross
Clepington Road
Dundee
DD3 8EA

Name
Address 1
Address 2
Address 3
Postcode

12345

Date: DD MM YY
Reference No: 123456789

Enquiries to: 0000 0000 000

Dear [Participant],

Thank you for taking the time to do the bowel screening test and for sending us your completed test.

Your personal risk of bowel cancer:

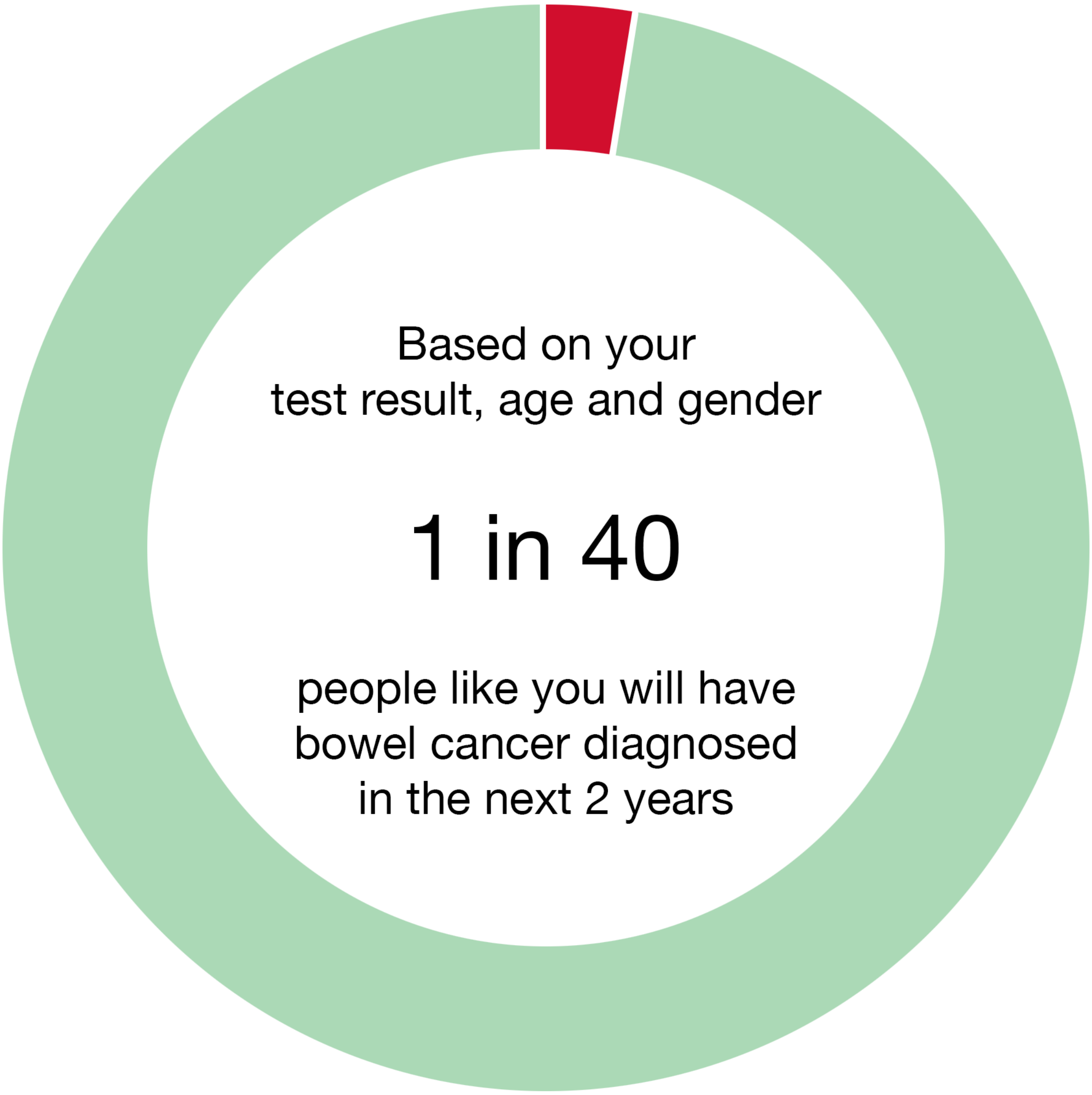

A further test called a ‘colonoscopy’ is the best way of checking for bowel cancer. A colonoscopy can find bowel cancer at the earliest stage of the disease, when it’s more treatable. It can also prevent cancer through the removal of polyps (small growths of cells on the bowel wall) during the test.

What’s a colonoscopy?

- It’s usually an outpatient appointment, so you shouldn’t need to stay in hospital for more than a few hours.
- A thin, flexible tube with a camera is used to examine the bowel. This means the doctor or nurse can fully examine the bowel.
- The tube reaches the bowel by passing through the bottom (back passage).

What happens now?

If you decide to have a colonoscopy and have bowel cancer found, you will be given an appointment to see a specialist as soon as possible to arrange treatment. Please read the Colonoscopy Information Booklet enclosed with this letter. This will further explain the colonoscopy to you, including how to prepare for the test and the risks involved. This will help you to decide whether or not you wish to proceed with the test.

If you decide not to have a colonoscopy, We will send you another test in two years’ time if you are still aged between 50 and 74. **It’s important that you do your bowel screening test every time you’re invited.** After that you can still request a test by contacting the Bowel Screening Centre.

Never ignore symptoms

It’s important to remember that while this test picks up most bowel cancer, it doesn’t find all bowel cancer. This is because the test looks for blood and not all cancers bleed all of the time. Look out for the following symptoms:

- Repeated bleeding from your bottom or blood in your poo
- A recent change in your bowel habit (how often you go to the toilet) that continues for more than four weeks without going back to normal
- Watery poo on its own or with constipation (constipation on its own is less likely to be serious)
- Severe pain in your stomach that won’t go away, especially after eating
- You’ve recently lost weight without trying
- You feel tired all the time and people keep telling you that you ‘look a bit pale’

Please also remember that changes can happen in between your bowel screening tests so please tell your GP if you notice any of these symptoms.

Yours sincerely,

Professor Bob Steele, Clinical Director, Scottish Bowel Screening Centre

Please see the next page to respond to questions about what you would intend to do in the event that you received this letter.

After you have read the Colonoscopy Information Booklet *(particularly the section on the risks/complications of colonoscopy on page 6), please answer the following questions:*

- Please indicate the extent to which you agree or disagree with the statement below **by ticking the appropriate box.**
- There is no right or wrong answer. We are interested in your personal views.

	Strongly agree	Agree	Slightly agree	Neither agree or disagree	Slightly disagree	Disagree	Strongly disagree
If I received information that my risk of bowel cancer was 1 in 40 then I would intend to have a colonoscopy.	☐	☐	☐	☐	☐	☐	☐
If I was told that I had a 1 in 40 chance of having bowel cancer, I would definitely choose to have a colonoscopy.	☐	☐	☐	☐	☐	☐	☐

If you were offered a colonoscopy following the test result mentioned in the letter, would you take up the offer?

Yes ☐

No ☐

Unsure ☐

Considering your decision whether or not to have a colonoscopy, **please tick the box below** which best describes your response to the following statements:

	Strongly agree	Agree	Slightly agree	Neither agree or disagree	Slightly disagree	Disagree	Strongly disagree
I know the decision available to me.	☐	☐	☐	☐	☐	☐	☐
I know the benefits of my decision.	☐	☐	☐	☐	☐	☐	☐
I know the risks and side effects of my decision.	☐	☐	☐	☐	☐	☐	☐

PRIVATE & CONFIDENTIAL

Name
Address 1
Address 2
Address 3
Postcode

12345

Scottish Bowel Screening Centre
Kings Cross
Clepington Road
Dundee
DD3 8EA

NHS SCOTLAND

Date: DD MM YY
Reference No: 123456789

Enquiries to: 0000 0000 000

Dear [Participant],

Thank you for taking the time to do the bowel screening test and for sending us your completed test.

Your personal risk of bowel cancer:

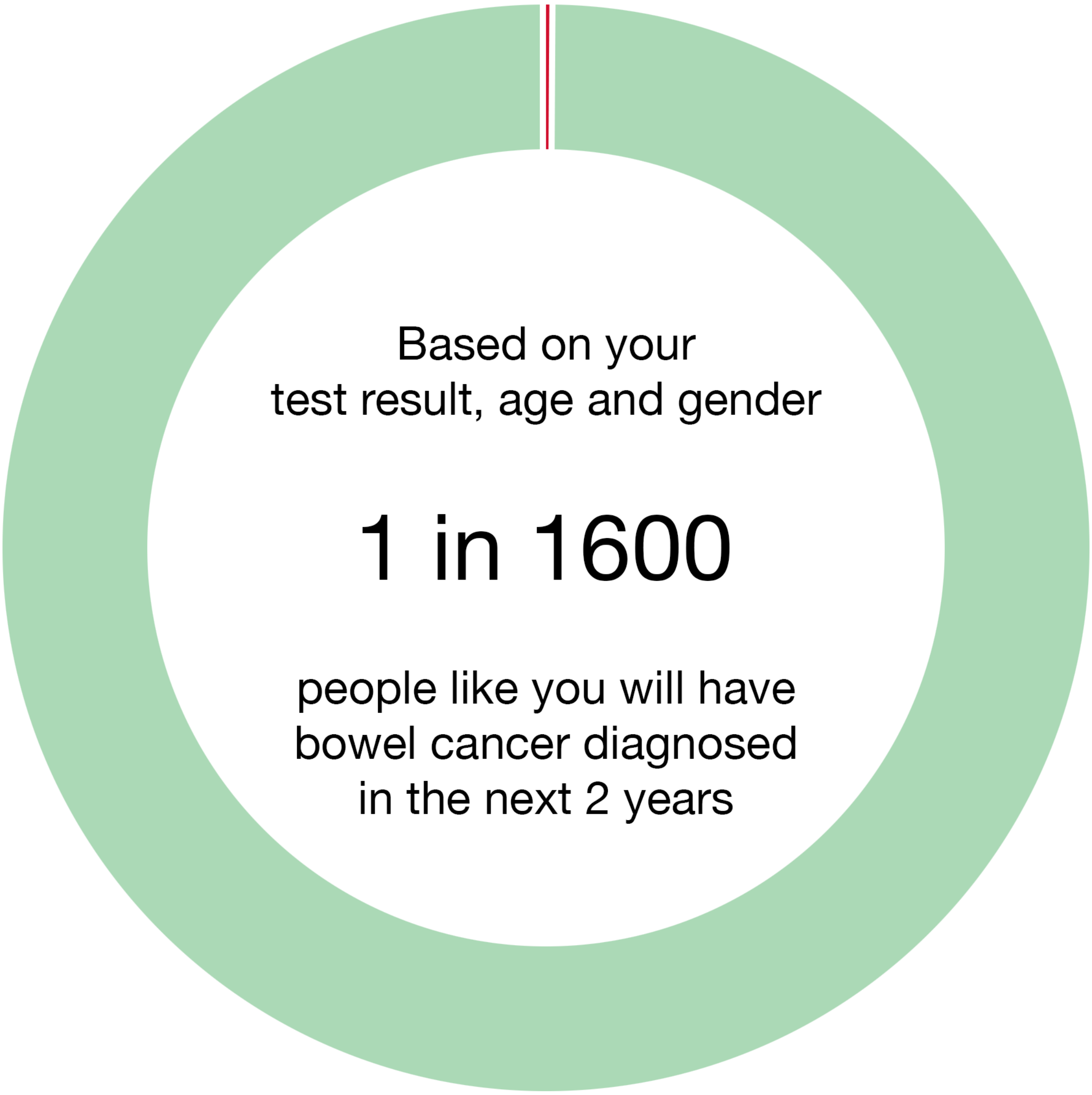

A further test called a ‘colonoscopy’ is the best way of checking for bowel cancer. A colonoscopy can find bowel cancer at the earliest stage of the disease, when it’s more treatable. It can also prevent cancer through the removal of polyps (small growths of cells on the bowel wall) during the test.

What’s a colonoscopy?

- It’s usually an outpatient appointment, so you shouldn’t need to stay in hospital for more than a few hours.
- A thin, flexible tube with a camera is used to examine the bowel. This means the doctor or nurse can fully examine the bowel.
- The tube reaches the bowel by passing through the bottom (back passage).

What happens now?

If you decide to have a colonoscopy and have bowel cancer found, you will be given an appointment to see a specialist as soon as possible to arrange treatment. Please read the Colonoscopy Information Booklet enclosed with this letter. This will further explain the colonoscopy to you, including how to prepare for the test and the risks involved. This will help you to decide whether or not you wish to proceed with the test.

If you decide not to have a colonoscopy, We will send you another test in two years’ time if you are still aged between 50 and 74. **It’s important that you do your bowel screening test every time you’re invited.** After that you can still request a test by contacting the Bowel Screening Centre.

Never ignore symptoms

It’s important to remember that while this test picks up most bowel cancer, it doesn’t find all bowel cancer. This is because the test looks for blood and not all cancers bleed all of the time. Look out for the following symptoms:

- Repeated bleeding from your bottom or blood in your poo
- A recent change in your bowel habit (how often you go to the toilet) that continues for more than four weeks without going back to normal
- Watery poo on its own or with constipation (constipation on its own is less likely to be serious)
- Severe pain in your stomach that won’t go away, especially after eating
- You’ve recently lost weight without trying
- You feel tired all the time and people keep telling you that you ‘look a bit pale’

Please also remember that changes can happen in between your bowel screening tests so please tell your GP if you notice any of these symptoms.

Yours sincerely,

Professor Bob Steele, Clinical Director, Scottish Bowel Screening Centre

Please see the next page to respond to questions about what you would intend to do in the event that you received this letter.

After you have read the Colonoscopy Information Booklet *(particularly the section on the risks/complications of colonoscopy on page 6), please answer the following questions:*

- Please indicate the extent to which you agree or disagree with the statement below **by ticking the appropriate box.**
- There is no right or wrong answer. We are interested in your personal views.

	Strongly agree	Agree	Slightly agree	Neither agree or disagree	Slightly disagree	Disagree	Strongly disagree
If I received information that my risk of bowel cancer was 1 in 1,600 then I would intend to have a colonoscopy.	☐	☐	☐	☐	☐	☐	☐
If I was told that I had a 1 in 1,600 chance of having bowel cancer, I would definitely choose to have a colonoscopy.	☐	☐	☐	☐	☐	☐	☐

If you were offered a colonoscopy following the test result mentioned in the letter, would you take up the offer?

Yes ☐

No ☐

Unsure ☐

Considering your decision whether or not to have a colonoscopy, **please tick the box below** which best describes your response to the following statements:

	Strongly agree	Agree	Slightly agree	Neither agree or disagree	Slightly disagree	Disagree	Strongly disagree
I know the decision available to me.	☐	☐	☐	☐	☐	☐	☐
I know the benefits of my decision.	☐	☐	☐	☐	☐	☐	☐
I know the risks and side effects of my decision.	☐	☐	☐	☐	☐	☐	☐

PRIVATE & CONFIDENTIAL

Name
Address 1
Address 2
Address 3
Postcode

12345

Scottish Bowel Screening Centre
Kings Cross
Clepington Road
Dundee
DD3 8EA

NHS SCOTLAND

Date: DD MM YY
Reference No: 123456789

Enquiries to: 0000 0000 000

Dear [Participant],

Thank you for taking the time to do the bowel screening test and for sending us your completed test.

Your personal risk of bowel cancer:

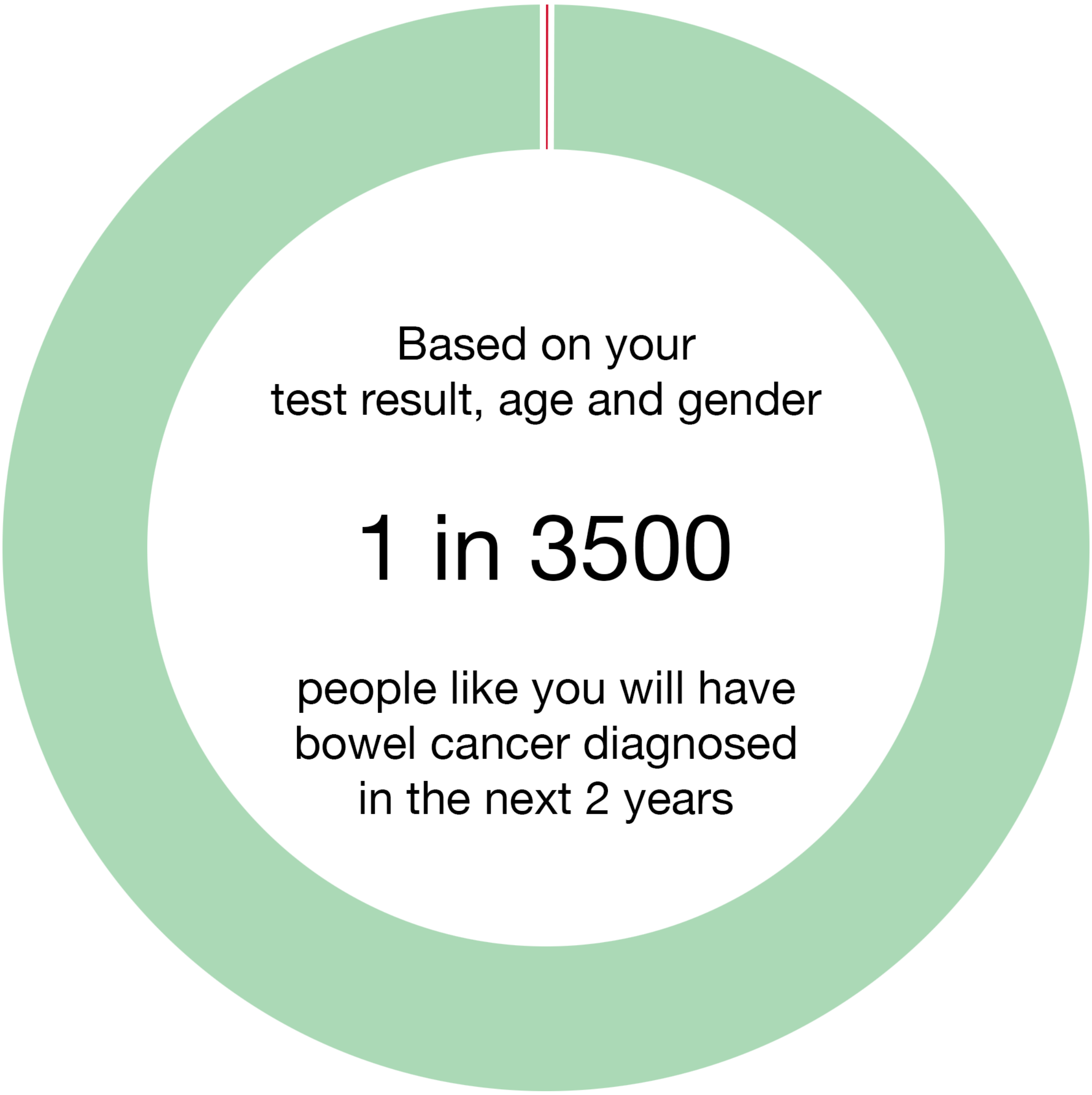

A further test called a ‘colonoscopy’ is the best way of checking for bowel cancer. A colonoscopy can find bowel cancer at the earliest stage of the disease, when it’s more treatable. It can also prevent cancer through the removal of polyps (small growths of cells on the bowel wall) during the test.

What’s a colonoscopy?

- It’s usually an outpatient appointment, so you shouldn’t need to stay in hospital for more than a few hours.
- A thin, flexible tube with a camera is used to examine the bowel. This means the doctor or nurse can fully examine the bowel.
- The tube reaches the bowel by passing through the bottom (back passage).

What happens now?

If you decide to have a colonoscopy and have bowel cancer found, you will be given an appointment to see a specialist as soon as possible to arrange treatment. Please read the Colonoscopy Information Booklet enclosed with this letter. This will further explain the colonoscopy to you, including how to prepare for the test and the risks involved. This will help you to decide whether or not you wish to proceed with the test.

If you decide not to have a colonoscopy, We will send you another test in two years’ time if you are still aged between 50 and 74. **It’s important that you do your bowel screening test every time you’re invited.** After that you can still request a test by contacting the Bowel Screening Centre.

Never ignore symptoms

It’s important to remember that while this test picks up most bowel cancer, it doesn’t find all bowel cancer. This is because the test looks for blood and not all cancers bleed all of the time. Look out for the following symptoms:

- Repeated bleeding from your bottom or blood in your poo
- A recent change in your bowel habit (how often you go to the toilet) that continues for more than four weeks without going back to normal
- Watery poo on its own or with constipation (constipation on its own is less likely to be serious)
- Severe pain in your stomach that won’t go away, especially after eating
- You’ve recently lost weight without trying
- You feel tired all the time and people keep telling you that you ‘look a bit pale’

Please also remember that changes can happen in between your bowel screening tests so please tell your GP if you notice any of these symptoms.

Yours sincerely,

Professor Bob Steele, Clinical Director, Scottish Bowel Screening Centre

Please see the next page to respond to questions about what you would intend to do in the event that you received this letter.

After you have read the Colonoscopy Information Booklet *(particularly the section on the risks/complications of colonoscopy on page 6), please answer the following questions:*

- Please indicate the extent to which you agree or disagree with the statement below **by ticking the appropriate box.**
- There is no right or wrong answer. We are interested in your personal views.

	Strongly agree	Agree	Slightly agree	Neither agree or disagree	Slightly disagree	Disagree	Strongly disagree
If I received information that my risk of bowel cancer was 1 in 3,500 then I would intend to have a colonoscopy.	☐	☐	☐	☐	☐	☐	☐
If I was told that I had a 1 in 3,500 chance of having bowel cancer, I would definitely choose to have a colonoscopy.	☐	☐	☐	☐	☐	☐	☐

If you were offered a colonoscopy following the test result mentioned in the letter, would you take up the offer?

Yes ☐

No ☐

Unsure ☐

Considering your decision whether or not to have a colonoscopy, **please tick the box below** which best describes your response to the following statements:

	Strongly agree	Agree	Slightly agree	Neither agree or disagree	Slightly disagree	Disagree	Strongly disagree
I know the decision available to me.	☐	☐	☐	☐	☐	☐	☐
I know the benefits of my decision.	☐	☐	☐	☐	☐	☐	☐
I know the risks and side effects of my decision.	☐	☐	☐	☐	☐	☐	☐
