# Supplementary material for: The impact of hypothetical PErsonalised Risk Information on informed choice and intention to undergo Colorectal Cancer screening colonoscopy in Scotland (PERICCS)—a randomised controlled trial
Source: BMC Med. 2020 Oct 20;18:285. doi: 10.1186/s12916-020-01750-3 (PMC7574531; doi:10.1186/s12916-020-01750-3)
Supplement: Supplementary file 3 — Additional file 3. Scenario letters booklet for control group (positive result letter) study arm; shows the current letter sent to Scottish Bowel Screening participants following a positive screening result. [file 12916_2020_1750_MOESM3_ESM.pdf]

STUDY ID: _ _ _ _

Scenario Letter

Impact of risk information in the Scottish Bowel Screening Programme

- In this booklet you will find a hypothetical bowel cancer screening result letter.
- The letter explains a positive bowel screening test result.
- Please answer the questions which follow the letter, then complete the separate Questionnaire Booklet.

University of Dundee   NHS SCOTLAND   UNIVERSITY of STIRLING

Scottish Bowel Screening Centre
Kings Cross
Clepington Road
Dundee
DD3 8EA

PRIVATE & CONFIDENTIAL

Name
Address 1
Address 2
Address 3
Postcode

12345

Date: DD MM YY
Reference No: 123456789

Enquiries to: 0000 0000 000

Dear [Participant],

Thank you for taking the time to do the bowel screening test and for sending us your completed test.

Your result:

The result of the test you provided shows that further investigation is required.

This result doesn't mean you have cancer but it does mean that we need to check on the cause of the bleeding. A further test called a 'colonoscopy' is the best way of looking for the cause of bleeding. In some, but not all, cases this may be due to bowel cancer. A colonoscopy can find bowel cancer at the earliest stage of the disease, when it's more treatable. It can also prevent cancer through the removal of polyps (small growths of cells on the bowel wall) during the test.

Very few of the people who need a colonoscopy will have bowel cancer, but some people will have bowel cancer found during a colonoscopy. If this happens you will be given an appointment to see a specialist as soon as possible to arrange treatment.

What's a colonoscopy?

- It's usually an outpatient appointment, so you shouldn't need to stay in hospital for more than a few hours.
- A thin, flexible tube with a camera will be used to examine your bowel. This means the doctor or nurse can fully examine your bowel.
- The tube will reach your bowel by passing through your bottom (back passage).

What happens now?

Please read the Colonoscopy Information Booklet enclosed with this letter. This will further explain the colonoscopy to you, including how to prepare for the test and the risks involved. This will help you to decide whether or not you wish to proceed with the test.

More information on what your bowel screening test result means can be found at:
www.nhsinform.scot/healthy-living/screening/bowel/bowel-screening

Yours sincerely,

Professor Bob Steele, Clinical Director, Scottish Bowel Screening Centre

Please turn over to respond to questions about what you would intend to do in the event that you received this letter.

We would now like you to think about whether or not you would have a colonoscopy, if you received this letter.

- Please indicate the extent to which you agree or disagree with the statement below **by ticking the appropriate box.**
- There is no right or wrong answer. We are interested in your personal views.

	Strongly agree	Agree	Slightly agree	Neither agree or disagree	Slightly disagree	Disagree	Strongly disagree
If I received information that the result of the test I provided showed that further investigation is required then I would intend to have a colonoscopy.	☐	☐	☐	☐	☐	☐	☐
If I was told that my screening test was abnormal meaning that there was a risk I had bowel cancer, I would definitely choose to have a colonoscopy.	☐	☐	☐	☐	☐	☐	☐

If you were offered a colonoscopy following the test result mentioned overleaf, would you take up the offer?

Yes ☐ No ☐ Unsure ☐

Considering your decision whether or not to have a colonoscopy **please tick the box below** which best describes your response to the following statements:

	Strongly agree	Agree	Slightly agree	Neither agree or disagree	Slightly disagree	Disagree	Strongly disagree
I know the decision available to me.	☐	☐	☐	☐	☐	☐	☐
I know the benefits of my decision.	☐	☐	☐	☐	☐	☐	☐
I know the risks and side effects of my decision.	☐	☐	☐	☐	☐	☐	☐
