# Supplementary material for: The impact of hypothetical PErsonalised Risk Information on informed choice and intention to undergo Colorectal Cancer screening colonoscopy in Scotland (PERICCS)—a randomised controlled trial
Source: BMC Med. 2020 Oct 20;18:285. doi: 10.1186/s12916-020-01750-3 (PMC7574531; doi:10.1186/s12916-020-01750-3)
Supplement: Supplementary file 4 — Additional file 4. Bowel Screening Test Booklet; the current leaflet provided to Scottish Bowel Screening Participants at the time of screening invitation to explain the benefits of participating in screening. [file 12916_2020_1750_MOESM4_ESM.pdf]

# This little test could help save your life.

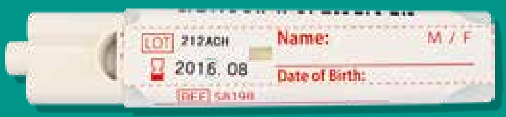


## The bowel screening test: What you need to know.

- One sample
- Complete at home
- Results within 2 weeks

Screening for life

NHS SCOTLAND

Healthier Scotland
Scottish Government

# Your bowel screening test

**Half a million people in Scotland do their bowel screening test each year.**

Bowel cancer is the third most common cancer in Scotland.

Your bowel screening test can find bowel cancer early when it can often be cured.

V14_28.4.17_6045

# **Do you know the benefits** of the bowel screening test?

9 out of 10 people survive bowel cancer if it's found and treated early

The test can find bowel cancer early, even when you and your GP don't know it's there

You do the test in the privacy of your own home

**The bowel screening test is now quicker and easier to use, with just one small sample to send.**

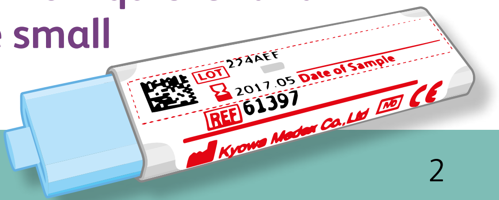

## Why am I being sent this bowel screening test?

From age 50 onwards, your risk of having bowel cancer is higher, and the early signs can be hidden. All men and women in Scotland aged 50 to 74 will be sent a bowel screening test. You can still take the test after you turn 75 if you'd like to – call the Bowel Screening Centre Helpline on **0800 0121 833** (textphone **18001 0800 0121 833**) and we'll send you a free test.

> **"You don't need to have symptoms. I didn't. I took the test and was shocked at the result but I am grateful I did. It saved my life"**
>
> Phyllis, 64, Lanark

## What if I have other health conditions?

It's safe to take the bowel screening test if you have other health conditions, including piles (haemorrhoids).

It's also OK to take the test if you're on medication, including blood thinners, iron tablets or antibiotics.

V14_28.4.17_6045

## Facts

- **You're 14 times more likely to survive bowel cancer if it's found early.**
- **4,000 people in Scotland get bowel cancer every year.**

## How to take the test

You can take the bowel screening test in the privacy of your own home in just a few steps. See the back of your letter for full details.

1. **Get ready:** take your label from the front of your letter and stick it on the side of the test marked with the +. Write the date you take the test on the other side.

2. **Do it:** twist the top of the test to open and take a small sample of your poo.

3. **Post it:** make sure you close the test. Put the finished test in the envelope and post it as soon as possible. It's ready to post once sealed inside the envelope provided. The envelope is designed to meet postal regulations and is safe to send. To protect postal workers, make sure the envelope is clean.

## What happens after I post my test?

We'll test your poo for hidden blood. Then we'll post your result to you two weeks after you send the test back to us.

## What happens after I get my result?

**Most people will be told their test is fine and they have no signs of bowel cancer.** But remember, changes can happen between one bowel screening test and the next. You should talk to your GP if you notice any of the changes listed on page 7.

We'll send you another test every two years until you turn 75.

## Does blood in my poo sample mean I definitely have bowel cancer?

**No.**

For every 500 people who take the test, only 10 will need to go for further testing. **Of these 10, only one will have bowel cancer.**

**If we find blood in your poo sample, we may need to do more to find out what's causing it**. We usually do this by using a colonoscopy. We'll write to tell you about this. The colonoscopy involves passing a thin, flexible tube into your bowel so it can be examined. It'll be done in hospital, but it usually takes less than an hour. You can have your colonoscopy and go home on the same day.

Hospital staff will tell you more about the colonoscopy and any risks for you.

V14_28.4.17_6045

> **My test went straight in the drawer. It seemed an embarrassing thing to do. Then I thought, I need to get over this! I did the test as I realised it could be lifesaving.**
>
> (Anne, 51, Glasgow)

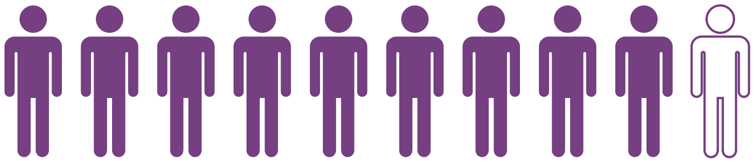

9 out of 10 people will survive if bowel cancer is found and treated early.

## Can the test find all bowel cancer?

**No**. The test will pick up most cases of bowel cancer but can't find them all. The test looks for hidden blood in your poo and not all cancers bleed. This is why as well as doing the test every time you are invited, it's also important to look out for symptoms of bowel cancer (listed below).

**Signs and symptoms:**

- Repeated bleeding from your bottom or blood in your poo.
- A recent change in your bowel habit (how often you go to the toilet) that continues for more than four weeks without going back to normal.
- Watery poo on its own or with constipation (constipation on its own is less likely to be serious).
- Severe pain in your stomach that won't go away, especially after eating.
- You've recently lost weight without trying.
- You feel tired all the time and people keep telling you that you 'look a bit pale'.

## Speak to your GP if …

you notice any of the changes above, or anything else that isn't normal for you. Changes can happen between one bowel screening test and the next.

# Can I do anything to reduce my risk of bowel cancer?

**Yes.**

Try:

- eating more foods that have lots of fibre, like wholemeal brown bread, wholegrain cereal and beans or pulses
- eating at least five portions of fruit and vegetables a day
- cutting down on processed and red meat (food like bacon, sausages or beef) – for information about healthy eating, visit **www.eatbetterfeelbetter.co.uk**
- getting some exercise most days of the week, even if it's just walking more
- keeping to a healthy weight
- drinking less alcohol – the less alcohol you drink the lower the risk – visit **www.alcohol-focus-scotland.org.uk** for more information
- stopping smoking – if you do smoke, call **Smokeline** on **0800 84 84 84** or visit **www.nhsinform.scot/smokeline** to help you quit.

# What happens to my details?

We keep a record of your tests and your results. All NHS staff must keep your personal health information confidential, which means they're not allowed to share it with anyone except you and other staff involved in your care.

We may use the information for research, education and training. But if we do, we'll remove any of your personal information.

We regularly review what we do to make sure we give you the best service possible. The information you give helps us to spot where we can improve.

But if you're not happy with your information being used in this way, call the helpline number above and let us know. You can also ask to see the information we hold about you.

The advisers at the Scottish Bowel Screening Centre will be glad to help with any questions you have and they'll respect your privacy. Contact the confidential helpline on
**0800 0121 833** (textphone **18001 0800 0121 833**)

V14_28.4.17_6045

## Where can I find more information or ask questions about the test?

The advisers at the Scottish Bowel Screening Centre will be happy to answer any questions you have and they'll respect your privacy.

- Phone the helpline on **0800 0121 833** (textphone **18001 0800 0121 833**)
- Email **bowelscreening.tayside@nhs.net**
- You can also go online and visit: **www.nhsinform.scot/bowelscreening**

You can watch a short film about how to do the test at **www.nhsinform.scot/bowelscreeningtest**

> **I just did the test. It was something that needed to be done. I'm so glad I did.**

(Charles. 65, Ayrshire)

**This leaflet tells you about the benefits of taking the bowel screening test.**

This resource may also be made available on request in the following formats:

translations | easy to read | audio | BSL | large print | braille

0131 314 5300

nhs.healthscotland-alternativeformats@nhs.net

Corporate member of Plain English Campaign
Committed to clearer communication
489

Published by NHS Health Scotland

1 South Gyle Crescent
Edinburgh EH12 9EB

© NHS Health Scotland 2016

ISBN: 978-1-84485-601-5

All rights reserved. Material contained in this publication may not be reproduced in whole or part without prior permission of NHS Health Scotland (or other copyright owners). While every effort is made to ensure that the information given here is accurate, no legal responsibility is accepted for any errors, omissions or misleading statements.

NHS Health Scotland is a WHO Collaborating Centre for Health Promotion and Public Health Development.

**www.healthscotland.scot**

6045 4/2017
