# Supplementary material for: The impact of hypothetical PErsonalised Risk Information on informed choice and intention to undergo Colorectal Cancer screening colonoscopy in Scotland (PERICCS)—a randomised controlled trial
Source: BMC Med. 2020 Oct 20;18:285. doi: 10.1186/s12916-020-01750-3 (PMC7574531; doi:10.1186/s12916-020-01750-3)
Supplement: Supplementary file 5 — Additional file 5. Colonoscopy Information Booklet; contains information to explain to participants what would happen before and during a colonoscopy and the risks and complications associated with the test. [file 12916_2020_1750_MOESM5_ESM.pdf]

Additional file 5. Colonoscopy Information Booklet.

Colonoscopy Information Booklet

Impact of risk information in the Scottish Bowel Screening Programme

- This booklet is to help you decide whether or not you would take up the offer of having a colonoscopy to check for bowel cancer.
- The booklet will tell you about what happens before and during a colonoscopy and the risks and complications associated with the test.
- Once you have read the information, please read through the bowel screening test result scenario letter/s provided and then answer the questions about your thoughts on having a colonoscopy.

University of Dundee NHS SCOTLAND UNIVERSITY of STIRLING

Personalised Risk Information Study, Colonoscopy Information Booklet, V1.0 03-10-18

What does this booklet include?

What is a colonoscopy? 3

What pain relief and sedation is available? 3

What about existing medical conditions? 4

What preparation is required for the test? 5

What happens during the procedure? 6

What are the risks and complications of having a colonoscopy? 7

What is a colonoscopy?

A colonoscopy allows direct inspection of the large bowel (rectum and colon). A slim flexible tube with a bright light at the end (a colonoscope) is passed through your anus into the rectum, and guided gradually around the colon and into the end of the small intestine.

Instruments can be passed through the colonoscope to allow samples to be taken and other procedures to be carried out. The appearance of your intestine may be recorded on a photograph for future reference.

How long does the colonoscopy take?

The colonoscopy takes from 20 minutes up to 45 minutes but may take longer. You will probably be in the department for 2 to 3 hours in total.

What pain relief and sedation is available?

Some people have Entonox for a colonoscopy. It is a gas you breathe in yourself from a mouthpiece or facemask and is designed to give pain relief without causing too much sleepiness. It is commonly used for pain during childbirth. Entonox works very quickly, wears off quickly and has few harmful effects. Entonox can make you nauseated, light headed or give you a dry mouth. This does not always occur, but if it does, you can stop breathing the gas and the feelings will go away almost immediately. Entonox can also make you feel very happy. This is why it is sometimes called laughing gas. Whilst this is not an unpleasant effect, some people may feel embarrassed. This effect also wears off very quickly after stopping inhaling the gas.

You will be monitored in the recovery area for at least 30 minutes after the test. Once you are assessed medically fit to be discharged you will be able

to drive and return to your normal activities. You will also be able to go home without an escort.

- If you are not suitable to have Entonox and decide to have sedation during the colonoscopy, you will have to organise an escort to collect you and stay with you for 24 hours. You cannot drive home alone. If you are having sedation a fine needle, a cannula, (like a drip) will be placed in a vein in your hand or arm so that a sedative injection can be given when you are in the Endoscopy room. Sedation is not a general anaesthetic - you will be awake and aware of the procedure. Sedation may make you forgetful; you may not remember details of the test. The effects of sedation last in your system for 24 hours.

What about existing medical conditions?

You will be given instructions about reducing or stopping the medication prior to your test if you take any of the following:

- medication for diabetes
- Clopidogrel/Plavix
- Warfarin
- Rivaroxaban (Xarelto)
- Dabigatran (Pradax)
- Apixaban

If you have Addison's disease, other arrangements can be made for you.

What preparation is required for the test?

It is essential that your bowel is completely empty on the day of the procedure so that the endoscopist can get a complete view unhampered by faeces. If you have a stoma, you may not need to take any bowel preparation.

Preparations for this need to start well beforehand, so it is important that you follow the instructions for your diet and take all of the bowel preparation, otherwise the procedure may need to be repeated.

7 days before the appointment you will need to:

- **Stop** taking any iron supplements or medicines that contain iron.
- **Stop or reduce,** if possible, painkillers containing codeine or dihydrocodeine. Paracetamol can be used as an alternative.
- **Stop** anti-diarrhoea medication for example Loperamide, Codeine Phosphate, Imodium, Lomotil, or stool bulking agents for example Fybogel, Regulan, Proctofibe.
- **Stop** eating seeded breads

You will be sent the bowel preparation purgative prior to your appointment along with instructions. The day before your appointment you should remain at home, take a clear fluid diet (no solids) and take the purgative supplied to you by mixing it with water and drinking 2-4 litres depending on the type of bowel preparation you receive.

Expect frequent loose or liquid bowel movements then loose motions to start within two hours, which will eventually become clear.
You will experience some urgency to go to the bathroom several times therefore stay near a toilet at this time. You may suffer nausea, colic, anal pain or headache during the day.

You will be asked to not eat anything on the day of your examination. You can drink clear fluids up to 2 hours before your test. You may eat and drink normally after the procedure.

What happens during the procedure?

You will be asked to lie on your left side. The endoscopist will examine your back passage gently with a gloved finger. The colonoscope will then be passed into the back passage. The instrument is then passed gradually round the rest of the colon. Small samples of tissues (biopsies) may need to be taken using forceps passed down the colonoscope. Polyps (small mushroom shaped protuberances of tissues) may be removed. If several polyps have to be removed, the scope may need to be passed several times.

Once the procedure is complete, the endoscopist will gradually withdraw the instrument, inspecting the bowel wall and sucking out as much air as possible.

During the procedure you may be aware of bloating and a sensation to move your bowels or pass wind. Do not feel inhibited from doing either: no stool is present, and this feeling is simply due to the presence of the scope. You may feel a "pushing" sensation inside, as the endoscopist presses round a corner. You may also feel the nurse pressing on your abdomen to guide the scope round the corner. A nurse will monitor you throughout the procedure. If biopsies are taken or polyps removed this does not cause any pain.

Sometimes despite bowel preparation there is too much faeces to complete the test. We will advise you of further tests or recommendations.

You are entitled to withdraw consent at any time, including during a procedure.

When the procedure is completed, you will be taken to the recovery room on the trolley. Your breathing, pulse and blood pressure will be recorded until you are fully recovered from the initial effects of the sedation, usually after 1 hour. You will be offered some refreshment and your cannula will be removed.

You may feel bloated for several hours, and may pass much wind or a little blood.

You may leave the hospital when your relative or friend is there to collect you.

What are the risks and complications of having a colonoscopy?

Colonoscopy is classified as an invasive investigation and because of that it has the possibility of associated complications. These occur extremely infrequently; we would wish to draw your attention to them and so with this information you can make your decision. The risks must be compared to the benefit of having the procedure carried out. The risks can be associated with the procedure itself and with administration of the sedation.

Risks of the colonoscopy examination

The risk of perforation or tear of the lining of the bowel is approximately 1 for every 2,000 examinations. An operation is nearly always required to repair the hole. The risk of perforation is higher with polyp removal (1 in 1,000).

Bleeding occurs at the site of polyp removal in approximately 1 for every 100-200 examinations where a polyp is removed. In the majority of cases, this is controlled at the time of endoscopy. Rarely, this may be severe and require further treatment in hospital.

Risks of sedation

Sedation can occasionally cause problems with breathing, heart rate and blood pressure. If any of these problems do occur, they are normally short lived. Careful monitoring by a fully trained endoscopy nurse ensures that any potential problems can be identified rapidly. Older patients and those who have significant health problems, for example, people with significant breathing difficulties due to a bad chest may be assessed by a doctor before being treated.

Is there any alternative to colonoscopy?

X ray tests such as barium enema or CT colonoscopy are available but these do not allow samples to be taken or polyps removed, and involve a significant amount of radiation.
