# Supplementary material for: The impact of hypothetical PErsonalised Risk Information on informed choice and intention to undergo Colorectal Cancer screening colonoscopy in Scotland (PERICCS)—a randomised controlled trial
Source: BMC Med. 2020 Oct 20;18:285. doi: 10.1186/s12916-020-01750-3 (PMC7574531; doi:10.1186/s12916-020-01750-3)
Supplement: Supplementary file 6 — Additional file 6. Questionnaire Booklet; contains some further questions on participants’ thoughts on having a colonoscopy and on the information provided to them. [file 12916_2020_1750_MOESM6_ESM.pdf]

Additional file 6. Questionnaire Booklet.

STUDY ID: _ _ _ _

Questionnaire Booklet

Impact of risk information in the Scottish Bowel Screening Programme

University of Dundee NHS SCOTLAND UNIVERSITY of STIRLING

Section 1

First, we would like you to think about the information you received about your risk of having bowel cancer.

- Please read the statements below and **tick the box** which best describes how you feel about the information you received.

	Strongly agree	Agree	Slightly agree	Neither agree or disagree	Slightly disagree	Disagree	Strongly disagree
I found the information easy to understand.	☐	☐	☐	☐	☐	☐	☐
I found the information presented distressing.	☐	☐	☐	☐	☐	☐	☐

We are very interested in your views on the information presented. Please make any additional comments below.

____

____

____

____

____

____

____

____

____

____

Section 2

A number of statements which people have used to describe themselves are given below. Read each statement and then **tick the most appropriate box to the right of the statement** to indicate how you feel **right now, at this moment.** There are no right or wrong answers. Do not spend too much time on any one statement but give the answer which seems to describe your present feelings best.

	Not at all	Somewhat	Moderately	Very much
I feel calm	☐	☐	☐	☐
I am tense	☐	☐	☐	☐
I feel upset	☐	☐	☐	☐
I am relaxed	☐	☐	☐	☐
I feel content	☐	☐	☐	☐
I am worried	☐	☐	☐	☐

Section 3

We would now like to ask you some questions about your knowledge of bowel cancer and bowel screening.

- Before answering the following questions, have you read the result letter scenario/s and the bowel screening and colonoscopy leaflets? Yes ☐ No ☐

Can people without symptoms have bowel cancer?

Yes ☐ **No** ☐

How many people out of 10 survive bowel cancer if treated early?

4 out of 10 ☐ **6 out of 10** ☐ **9 out of 10** ☐

Do you think that all cancers bleed, meaning that bowel screening test will find every cancer?

Yes ☐ **No** ☐

The bowel preparations required before a colonoscopy will mean you will experience urgency to go to a bathroom and you will need to remain at home.

True ☐ **False** ☐

How long does a colonoscopy usually take?

5-10 minutes ☐ **20-45 minutes** ☐ **1-2 hours** ☐

How many people who have a colonoscopy will experience bowel perforation (tear of the lining of the bowel)?

1 in every 2000 ☐ **1 in every 500** ☐ **1 in every 200** ☐

Bleeding occurs with approximately one in every 100-200 polyp removals.

True ☐ **False** ☐

The sedation used during colonoscopy occasionally cause problems with breathing, heart rate and blood pressure?

True ☐ **False** ☐

Section 4

We would now like you to think about how you think you would feel if you were invited to have a colonoscopy.

- First of all, please tick the appropriate box to indicate whether or not you have had a colonoscopy before:

Yes ☐

No ☐

Unsure ☐

- Now, please indicate the extent to which you agree or disagree with the statements below **by ticking the appropriate box.**
- There are no right or wrong answers. We are interested in your personal views.

**YOUR BELIEFS ABOUT HAVING A COLONOSCOPY**	Strongly disagree	Disagree	Uncertain	Agree	Strongly agree
I would choose to have a colonoscopy because it would reduce my risk of dying from bowel cancer.					
I would choose to not have a colonoscopy because it can be uncomfortable.					
I would choose to have a colonoscopy because the benefits outweigh the risks.					
I would expect a colonoscopy to be very distressing.					
I would expect a colonoscopy to be only mildly uncomfortable.					
I would choose not to have a colonoscopy as I would expect the bowel preparation to be unpleasant.					
I would choose not to have a colonoscopy as I would find it embarrassing.					
I would choose not to have a colonoscopy as it would make me feel anxious.					
Having to stop taking some medications would put me off having a colonoscopy.					

Thank you very much for completing these questionnaires. Your answers are completely confidential. The answers you have given will be very valuable to our research, and will help us understand how people respond to information about their risk of having bowel cancer.

Thank you very much for taking part in our research.
