# Supplementary material for: The impact of hypothetical PErsonalised Risk Information on informed choice and intention to undergo Colorectal Cancer screening colonoscopy in Scotland (PERICCS)—a randomised controlled trial
Source: BMC Med. 2020 Oct 20;18:285. doi: 10.1186/s12916-020-01750-3 (PMC7574531; doi:10.1186/s12916-020-01750-3)
Supplement: Supplementary file 7 — Additional file 7. Internal reliability of study measures; contains the results of statistical tests to measure the internal reliability of several of the study measures. [file 12916_2020_1750_MOESM7_ESM.pdf]

Internal reliability of PERICCS study measures

Internal reliability was assessed via Cronbach's alpha. Reliability was good for the 9-item attitude scale ($\alpha = 0.79$) and for the 3 items regarding intention to take up colonoscopy for high ($\alpha = 0.77$), moderate ($\alpha = 0.86$), and low risk ($\alpha = 0.90$) categories. Reliability was excellent for the 3 Decisional Support items for high ($\alpha = 0.88$), moderate ($\alpha = 0.89$), and low risk ($\alpha = 0.91$) options across all treatment arms. Internal reliability for the knowledge questions was less good ($\alpha = 0.52$), possibly reflecting the fact that these 8 items covered diverse questions measuring knowledge and risk of both colorectal cancer and colonoscopy. Nonetheless the validity of the knowledge scale was supported by its relationship to other variables i.e. higher knowledge was related to more positive attitudes towards colonoscopy ($r = 0.26$, $p < 0.001$), as well as higher intention to take up colonoscopy in the high and moderate risk groups only (high risk: $r = 0.20$, $p = 0.001$; moderate risk: $r = 0.17$, $p = 0.024$; low risk: $r = 0.10$, $p = 0.186$). Higher knowledge was also related to less decisional conflict in all risk categories (high risk: $r = 0.30$, $p < 0.001$; moderate risk: $r = 0.33$, $p < 0.001$; low risk: $r = 0.23$, $p = 0.002$) and to lower anxiety ($r = -0.13$, $p = 0.025$). Similarly, the validity of the attitudes scale was demonstrated by the fact that more positive attitudes towards colonoscopy were related to higher intention to take up colonoscopy (high risk: $r = 0.54$, $p < 0.001$; moderate risk: $r = 0.44$, $p < 0.001$; low risk: $r = 0.34$, $p < 0.001$) and less decisional conflict (high risk: $r = 0.43$, $p < 0.001$; moderate risk: $r = 0.51$, $p < 0.001$; low risk: $r = 0.39$, $p < 0.001$). More positive attitudes were also related to lower anxiety ($r = -0.21$, $p < 0.001$).
